# Microbial community structure analysis in *Acer palmatum* bark and isolation of novel bacteria IAD-21 of the candidate division FBP

**DOI:** 10.7717/peerj.7876

**Published:** 2019-10-29

**Authors:** Kazuki Kobayashi, Hideki Aoyagi

**Affiliations:** 1 Division of Life Sciences and Bioengineering, Graduate School of Life and Environmental Sciences, University of Tsukuba, Tsukuba, Ibaraki, Japan; 2 Faculty of Life and Environmental Sciences, University of Tsukuba, Tsukuba, Ibaraki, Japan

**Keywords:** Tree bark, Microbial community, Uncultured bacteria, Candidate division FBP

## Abstract

**Background:**

The potential of unidentified microorganisms for academic and other applications is limitless. Plants have diverse microbial communities associated with their biomes. However, few studies have focused on the microbial community structure relevant to tree bark.

**Methods:**

In this report, the microbial community structure of bark from the broad-leaved tree *Acer palmatum* was analyzed. Both a culture-independent approach using polymerase chain reaction (PCR) amplification and next generation sequencing, and bacterial isolation and sequence-based identification methods were used to explore the bark sample as a source of previously uncultured microorganisms. Molecular phylogenetic analyses based on PCR-amplified 16S rDNA sequences were performed.

**Results:**

At the phylum level, *Proteobacteria* and *Bacteroidetes* were relatively abundant in the *A. palmatum* bark. In addition, microorganisms from the phyla *Acidobacteria*, *Gemmatimonadetes*, *Verrucomicrobia*, *Armatimonadetes*, and candidate division FBP, which contain many uncultured microbial species, existed in the *A. palmatum* bark. Of the 30 genera present at relatively high abundance in the bark, some genera belonging to the phyla mentioned were detected. A total of 70 isolates could be isolated and cultured using the low-nutrient agar media DR2A and PE03. Strains belonging to the phylum *Actinobacteria* were isolated most frequently. In addition, the newly identified bacterial strain IAP-33, presumed to belong to *Acidobacteria*, was isolated on PE03 medium. Of the isolated bacteria, 44 strains demonstrated less than 97% 16S rDNA sequence-similarity with type strains. Molecular phylogenetic analysis of IAD-21 showed the lowest similarity (79%), and analyses suggested it belongs to candidate division FBP. Culture of the strain IAD-21 was deposited in Japan Collection of Microorganisms (JCM) and Deutsche Sammlung von Mikroorganismen und Zellkulturen (DSMZ) as JCM 32665 and DSM 108248, respectively.

**Discussion:**

Our results suggest that a variety of uncultured microorganisms exist in *A. palmatum* bark. Microorganisms acquirable from the bark may prove valuable for academic pursuits, such as studying microbial ecology, and the bark might be a promising source of uncultured bacterial isolates.

## Introduction

The total number of microorganisms existing on the earth is speculated to range from 10^29^ to 10^30^ organisms (Whitman, Coleman & Wiebe, 1998; Kallmeyer et al., 2012). It is reported that the number of operational taxonomic units (OTUs) detected from one g of soil is up to 52,000 (Roesch et al., 2007). The number of bacterial species that currently have been isolated, investigated with regards to physiological properties, and assigned scientific names is about 15,000 (Parte, 2018). This is only 1% of the total number of bacterial species presumed to exist on earth, and the remaining 99% of uncultured microorganisms is called the “microbial dark matter” (Ledford, 2015). Until now, only cultivable microorganisms among the 1% have been used to comprehend the overall microbial ecosystem and identify novel useful genes, but the exploration of these cultivable microbes have reached a plateau in recent years (Puspita et al., 2012). Since the microbial dark matter is expected to potentially impact the current status of academic and industrial fields, comprehensive environmental genome analyses are being conducted around the world (Rinke et al., 2013; McCalley et al., 2014). However, unraveling microbial functions, which cannot be elucidated from the nucleotide sequence alone, or the practical utilization of uncultured microorganisms, requires pure culture isolation and cultivation (Stewart, 2012). Since cultivation of the remaining 99% of microorganisms holds great potential, exploration and isolation of microorganisms from various environments are desirable.

Numerous analyses on symbiotic microorganisms have been conducted for many terrestrial plants, and their microbial community structures are determined not only by plant species, but also by factors such as plant organs and environmental factors (Wieland, Neumann & Backhaus, 2001; Schlaeppi et al., 2010; Edwards et al., 2015; Zarraonaindia et al., 2015). Many of these microorganisms provide benefits to plants, such as the promotion of plant growth (Köberl et al., 2013), modification of plant-hormone production (Bodenhausen et al., 2014), and resistance to disease (Berendsen, Pieterse & Bakker, 2012). To understand the plant-microbial symbiotic relationship and its impact on the ecosystem, comprehensive analysis of the plant symbiotic microbial community structure and further isolation of symbiotic microorganisms, including uncultured microorganisms, are necessary. For example, in the case of agricultural crops and model plants, including *Arabidopsis thaliana*, exhaustive analyses of symbiotic microorganisms’ function and community structure have been performed using both culture-independent and culture-dependent methods (Delmotte et al., 2009; Manter et al., 2010; Vorholt, 2012; Comby et al., 2016). However, for some plant types, the exploration of microbial resources has not been sufficiently conducted yet. Tanaka et al. (2012) focused on the rhizosphere of aquatic plants, which have not been thoroughly investigated for studying symbiotic microbial communities, and isolated *Armatimonas rosea* YO-36^T^ (Former Candidate division OP10) from the roots of *Phragmites australis*. Tanaka et al. (2017) also investigated the roots of the aquatic plants *Iris pseudacorus* and *Scirpus juncoides*, and isolated microorganisms belonging to *Acidobacteria* and *Verrucomicrobia*, which are relatively difficult to cultivate. Microorganisms isolated from such aquatic plants are relatively novel, even if they belong to taxa with high cultivation frequency. Since environmental samples that have not been explored thus far lack information on microbial communities and isolates in databases, it is suggested that the novelty of cultured microorganisms is necessarily high from such unexplored potential microbial resources.

Analyses of the structures of microbial communities present on trees have been previously conducted (Mocali et al., 2003; Moore et al., 2006; Taghavi et al., 2009; Redford et al., 2010; Filteau et al., 2010; Laforest-Lapointe, Messier & Kembel, 2016a, 2016b). However, very few studies have focused on the tree bark. The bark refers to the outer side of the cambium surrounding the xylem of the tree and is composed of an inner bark, which is the living tissue consisting of phloem, and an outer bark, which is the dead tissue of the cork. The bark is composed of polysaccharides (cellulose, hemicellulose), pectin substances, phenolic polymers such as lignin and high molecular weight tannins, and cross-linked polyesters such as suberin and cutin. The bark contains greater amounts of extracts (polyphenol and suberin), minerals, and lignin than the center of the tree (Feng et al., 2013). As a protective tissue, the bark consists of compounds that are resistant to microbial degradation, such as suberin (Baldrian, 2017). In addition, the bark is impregnated with resin that inhibits the growth of microorganisms (Baldrian, 2017). The bark protects the cambium from precipitation, heat, frost, and UV radiation and acts as a barrier against the attack of bacteria, fungi, parasitic plants, insects, and animals (Sakai, 2001). By adapting to tree bark, microorganisms may be able to acquire a stable habitat. In the case of bark (especially old bark), the tree canopy blocks precipitation and UV irradiation, and there is less disturbance than in other tissues such as leaves and branches, suggesting that microorganisms can stably inhabit areas for a long time (Leff et al., 2015). Further, microorganisms can colonize microsites such as cracks and lenticels, which represent a more favorable environment for microbial growth because they retain humidity and nutrients (Buck, Lachance & Traquair, 1998), and the symbiotic microorganisms can utilize plant biomass and photosynthetic products as carbon sources in such a stable habitat. Therefore, the bark presents a suitable habitat for slow-growing microbes and those susceptible to disturbance. However, compared with other tissues such as leaves and rhizosphere, microbial community structure analysis, and isolation of microorganisms (especially bacteria) including uncultured microorganisms from the bark have not been sufficiently performed.

Shen & Fulthorpe (2015) revealed the differences among the microbial community structures within the tree branches of the species *Acer negundo*, *Ulmus pumila*, and *U. parvifolia*, using isolation of the microorganisms and various culture-independent analyses. Ulrich, Ulrich & Ewald (2007) demonstrated the impact of different hybrid poplar clones on the endophytic community structure in branches and leaves using terminal restriction fragment length polymorphism analysis and analyzed the microbial community structure within the branches and leaves of poplar trees using isolation of the microorganisms and clone analysis. However, these two studies used tree branches as the source material, where the environment is completely different from that of the bark. In addition, Aschenbrenner et al. (2017) revealed the microbial community structure of *Acer pseudoplatanus* bark, symbiotic moss, and lichens using next generation sequencing. Interestingly, there are a few studies that suggested the bark microbial community is different from that in other organs. Martins et al. (2013) investigated the cultivation and isolation of microorganisms from grapevine and reported that bacterial genera obtained from the bark differed from those obtained from the fruits and leaves. Leff et al. (2015) conducted a culture-independent analysis of *Ginkgo biloba* bark, branches, young branches, and leaves using high throughput 454 pyrosequencing and showed that the diversity of microbial communities in the old bark was the highest. In addition, phyla containing bacterial species that are generally difficult to culture, or are uncultured, such as *Acidobacteria*, *Armatimonadetes*, and the candidate division WYO (Serkebaeva et al., 2013; Weiss et al., 2015), were detected in higher proportions in the bark than in other tree organs. However, since there are few reported cases of analysis of the microbial community structure of bark samples and the isolation (acquisition) of uncultured microorganisms at higher taxonomic levels such as the phylum or class level, there is insufficient evidence to deduce whether bark is an excellent source of uncultured microorganisms.

Nonetheless, previous observations suggested that tree bark may harbor special microbial communities and that isolation and analysis of microorganisms from bark may provide insights into unknown microbial ecosystems and tree-microbial symbiosis. In the current study, we targeted the bark of *Acer palmatum*, which is a deciduous broad-leaved tree widely growing in Japan, and analyzed the microbial community structure using MiSeq-based next generation sequencing. In addition, we attempted to isolate and cultivate microorganisms by standard methods using low-nutrient agar media.

## Materials and Methods

### Sample collection and pre-treatment

Bark sample was collected from mature *Acer palmatum* from the Ichimura Foundation for New Technology Botanical Research Gardens, Atami, Shizuoka, Japan (35.107336 N, 139.047729 E) using sterile tweezers and scissors. *Acer palmatum* trees used in this study were at least 80 years old or more and naturally occurring. The botanical research garden is 277–310 m above sea level, along a gentle slope facing south-south-east. Although the botanical garden is an artificially landscaped Japanese garden with artificially planted plants, it also contains several natural plants. A sample for culture-independent analysis was collected in November 2015, and a bark sample for microbial isolation was collected in June 2016. Bark samples for both analyses were collected from the same position (at a height of 1.0–1.5 m) on the same single tree. Further, in order to re-analyze the microbial community structure by culture-independent analysis, we collected three samples (at heights of 1.0, 1.5, and 2.0 m) from each of two individual trees (one being the previously analyzed tree) in February 2018. Approximately 4.7 g of bark fragments from the surface to a depth of about two mm were collected. Since the thickness of the bark (phloem and periderm) of another member of the same genus, *Acer rubrum*, is 0.8 ± 0.03 mm (Hammond et al., 2015), it was considered that the bark area should be covered by this sampling and that the collected sample contains both epiphyte and endophyte in this range. The collected bark samples were minced using sterilized tweezers and scissors, suspended in 40 ml of phosphate-buffered saline, thoroughly mixed by vortexing, and sonicated at 42 kHz for 3 min using a Bransonic Ultrasonic Cleaner 3510J-DTH (Branson Ultrasonic Corporation, Danbury, CT, USA) to detach the microorganisms adhering to the bark surface. The microbial suspensions from the bark samples were stored at −80 °C in 10% (v/v) glycerol.

### Culture-independent analysis

To assess the structure of the microbial community in the bark of *Acer palmatum*, 16S amplicon sequencing using MiSeq was performed. From the above bark suspension, five ml of the supernatant was collected by pipetting so as to minimize contamination of the bark fragments, and DNA was extracted using a Fast DNA Spin Kit (MP Biomedicals, LCC, Santa Ana, CA, USA) according to the manufacturer’s instructions. The extracted DNA was adjusted with distilled water to a concentration of 30 ng/μl in a total volume of 50 μl. The DNA concentration was fluorometrically determined using Qubit Assay Kits (Thermo Fisher Scientific Inc., Waltham, MA, USA) and a Nanophotometer (Implen GmbH, Munich, Germany). Sequence analysis of the bark samples using a MiSeq system (Illumina, Inc., San Diego, CA, USA) was performed by Fasmac Co., Ltd (Atsugi, Japan). During the first round of polymerase chain reaction (PCR) amplification, template DNA was amplified using a primer set targeting the V4 region of 16S rDNA. The hot-start PCR reaction consisted of five ng of the starting template, 10 µM of the forward primer 1st_PCR_515F (5′-ACA CTC TTT CCC TAC ACG ACG CTC TTC CGA TCT—[GTG CCA GCM GCC GCG GTA A]-3′) and the reverse primer 1st_PCR_806R (5′-GTG ACT GGA GTT CAG ACG TGT GCT CTT CCG ATC T—[GGA CTA CHV GGG TWT CTA AT]-3′), 0.2 µl of ExTaq HS polymerase (Takara Bio Inc., Kusatsu, Japan), 1.6 µl of dNTPs, and two µl of 10× Ex Taq buffer in a total reaction volume of 20 μl. The first PCR primers included the adapter sequences for the second PCR and sequences homologous to the V4 region of 16S, as shown in parentheses. The thermal cycling profile included an initial denaturing cycle of 94 °C for 30 s, followed by 20 sequential cycles of 94 °C for 15 s, 50 °C for 30 s, 72 °C for 30 s, and a final extension period of 72 °C for 5 min, ending with a hold cycle at 4 °C. The PCR products were purified using an Agencourt AMPure XP Kit (Beckman Coulter, Inc., Brea, CA, USA) using the manufacturer’s instructions. The second PCR reaction included two μl of the purified template DNA, 10 µM of the forward primer 2nd_F (5′-[AAT GAT ACG GCG ACC ACC GAG ATC TAC AC]—[XXXXXXXX]—[ACA CTC TTT CCC TAC ACG ACG C]-3′) and the reverse primer 2nd_R (5′-[CAA GCA GAA GAC GGC ATA CGA GAT]—[YYYYYYYY]—[GTG ACT GGA GTT CAG ACG TGT G]-3′), 0.2 µl of Ex Taq HS polymerase (Takara, Kusatsu, Japan), 1.6 µl of dNTPs, and two µl of 10× Ex Taq buffer in a total reaction volume of 20 µl. The second PCR primers included the following sequences: 5′—[flow cell binding region]—[Illumina i5/i7 index]—[primer binding region (homologous to the 1st primer sequence)]—3′. The thermal cycling profile for the second PCR was a single cycle of 94 °C for 2 min, followed by eight cycles of 94 °C for 10 s, 60 °C for 30 s, 72 °C for 30 s, with one final cycle of 72 °C for 5 min, and a hold cycle of 4 °C. The products from the second PCR were purified using an Agencourt AMPure XP Kit. The DNA concentrations were determined using Qubit Assay Kits, and the PCR amplicons were mixed and subjected to 2 × 250 bp paired-end sequencing using MiSeq System v2. Cluster formation was performed using MiSeq Reagent Kit v2 and PhiX Control Kit v3, and sequence analysis was performed using MiSeq Control Software ver 2.4.1.3, Real Time Analysis ver 1.18.54 and bcl2fastq ver 1.8.4.

Analysis of the sequencing results included trimming of the primer region using Fastx toolkit, version 0.0.13.2 (Gordon & Hannon, 2010), joining of the forward and reverse reads using FLASH, version 1.2.10 (Magoč & Salzberg, 2011), and quality filtering with sickle tool, version 1.33 (Joshi & Fass, 2011). Filtering of the raw sequence reads was performed based on the following criteria: (1) the start region of both reads exactly matched the primer of the V4 region; (2) the minimum length was 40 bp, after the trimming of the primer region and the low-quality sequence; and (3) both reads could be joined, and the length after joining was 246–260 bp (amplicon sequence length was 285–299 bp). The 97% identity OTU clustering and chimera filtering were performed using UCHIME (USEARCH package v8.0.1623) (Edgar et al., 2011) in QIIME, version 1.9.0 (Caporaso et al., 2010). These data were then used to assign taxonomy against the Greengenes 13_8 database (DeSantis et al., 2006) with a 97% similarity threshold using the UCLUST v1.2.22q (Edgar, 2010) in the assign taxonomy script of QIIME. Details of commands and parameters are summarized in Table S1.

### Isolation of bacteria

In order to examine whether previously uncultivated microorganisms could be acquired from the bark of *Acer palmatum*, cultivation was performed using a general low-nutrient agar plate medium. Bark-suspension supernatants (100 μl) were 10-fold serially diluted (10–10^3^ fold) and were inoculated into Reasoner’s 2A (R2A; Wako Pure Chemical Industries, Ltd., Osaka, Japan) culture medium that had been 10-fold diluted (DR2A) and PE03 medium (Tamaki et al., 2005), and incubated at 25 °C for 2 weeks under dark conditions. The strains isolated from PE03 medium are represented as Strain No. IAP and the strains isolated from DR2A medium are represented as Strain No. IAD as shown in Tables 1 and 2, respectively. In order to selectively isolate slow-growing microbes, small colonies that were visible but less than one mm in diameter were targeted. For each medium, 48 colonies were isolated based on colony color and shape. Isolated colonies were suspended in 20 μl of Tris-EDTA (TE) buffer (Sigma-Aldrich Co. LCC, St. Louis, MO, USA) for DNA extraction, in addition to preparing one ml of glycerol stock (five mM Mops, 10% (v/v) Glycerol, pH 7.0) of each isolate. The DNA samples were stored at −20 °C, and the glycerol stocks at −80 °C.

**Table 1:** Most similar sequences of isolated microbes from PE03 medium.

Strain no.	Phylum or class	Most similar sequence	Accession no.	Similarity (%)
IAP-1	*Alphaproteobacteria*	*Bradyrhizobium embrapense* strain SEMIA 6208	AY904773	100
IAP-2	*Actinobacteria*	*Mycobacterium peregrinum* strain ATCC 14467	AF058712	97
IAP-3	*Bacteroidetes*	*Hymenobacter terrae* strain DG7A	KF862488	93
IAP-4	*Actinobacteria*	*Amnibacterium soli* strain PB243	KC251736	98
IAP-5	*Actinobacteria*	*Amnibacterium soli* strain PB243	KC251736	96
IAP-7	*Gammaproteobacteria*	*Moraxella osloensis* strain DSM 6998	AB643599	99
IAP-8	*Bacteroidetes*	*Spirosoma spitsbergense* strain SPM-9	EF451725	92
IAP-9	*Alphaproteobacteria*	*Sphingomonas mucosissima* strain CP173-2	AM229669	99
IAP-10	*Actinobacteria*	*Actinomycetospora chibensis* strain TT04-21	AB514517	98
IAP-11	*Alphaproteobacteria*	*Psychroglaciecola arctica* strain M6-76	KC511070	95
IAP-12	*Actinobacteria*	*Actinomycetospora chlora* strain TT07I-57	AB514519	97
IAP-14	*Alphaproteobacteria*	*Afipia birgiae* strain 34632	AF288304	99
IAP-15	*Alphaproteobacteria*	*Sphingomonas mucosissima* strain CP173-2	AM229669	99
IAP-16	*Alphaproteobacteria*	*Sphingomonas asaccharolytica* strain Y-345	Y09639	99
IAP-17	*Actinobacteria*	*Pseudonocardia endophytica* strain YIM 56035	DQ887489	96
IAP-18	*Alphaproteobacteria*	*Novosphingobium barchaimii* strain LL02	JN695619	98
IAP-19	*Alphaproteobacteria*	*Novosphingobium barchaimii* strain LL02	JN695619	98
IAP-20	*Alphaproteobacteria*	*Sphingomonas hankookensis* strain ODN7	FJ194436	98
IAP-21	*Actinobacteria*	*Microbacterium fluvii* strain YSL3-15	AB286028	97
IAP-23	*Actinobacteria*	*Cellulomonas pakistanensis* strain NCCP-11	AB618146	97
IAP-24	*Actinobacteria*	*Cellulomonas pakistanensis* strain NCCP-11	AB618146	97
IAP-27	*Betaproteobacteria*	*Variovorax paradoxus* strain NBRC 15149	AB680784	99
IAP-28	*Betaproteobacteria*	*Variovorax guangxiensis* strain GXGD002	JF495126	99
IAP-29	*Actinobacteria*	*Jatrophihabitans huperziae* strain I13A-01604	KR184574	91
IAP-30	*Actinobacteria*	*Lysinimonas soli* strain SGM3-12	JN378395	98
IAP-31	*Actinobacteria*	*Amnibacterium kyonggiense* strain KSL51201-037	FJ527819	96
IAP-32	*Alphaproteobacteria*	*Phenylobacterium aquaticum* strain W2-3-4	KT309087	94
IAP-33	*Acidobacteria*	*Terriglobus roseus* strain KBS 63	DQ660892	99
IAP-35	*Actinobacteria*	*Microbacterium saccharophilum* strain K-1	AB736273	96
IAP-36	*Alphaproteobacteria*	*Sphingomonas koreensis* strain NBRC 16723	AB681117	98
IAP-37	*Bacteroidetes*	*Flavobacterium rivuli* strain WB3.3-2	AM934661	93
IAP-39	*Actinobacteria*	*Microbacterium saccharophilum* strain K-1	AB736273	97
IAP-40	*Alphaproteobacteria*	*Brevundimonas albigilva* strain NHI-13	KC733808	95
IAP-41	*Actinobacteria*	*Microlunatus panaciterrae* strain Gsoil 954	AB271051	96
IAP-42	*Actinobacteria*	*Microbacterium saccharophilum* strain K-1	AB736273	96
IAP-45	*Bacteroidetes*	*Mucilaginibacter rigui* strain NBRC 101115	AB681382	96
IAP-46	*Actinobacteria*	*Nakamurella multipartita* strain DSM 44233	CP001737	94
IAP-47	*Actinobacteria*	*Microbacterium fluvii* strain YSL3-15	AB286028	97
IAP-48	*Alphaproteobacteria*	*Sphingomonas asaccharolytica* strain Y-345	Y09639	98

**Table 2:** Most similar sequences of isolated microbes from DR2A medium.

Strain no.	Phylum or class	Most similar sequence	Accession no.	Similarity (%)
IAD-1	*Bacteroidetes*	*Mucilaginibacter rigui* strain NBRC 101115	AB681382	96
IAD-2	*Actinobacteria*	*Actinomycetospora cinnamomea* strain IY07-53	AB514520	97
IAD-3	*Bacteroidetes*	*Spirosoma panaciterrae* strain Gsoil 1519	EU370956	90
IAD-4	*Actinobacteria*	*Microbacterium fluvii* strain YSL3-15	AB286028	97
IAD-5	*Bacteroidetes*	*Spirosoma spitsbergense* strain SPM-9	EF451725	90
IAD-6	*Actinobacteria*	*Nocardioides islandensis* strain MSL 26	EF466123	99
IAD-7	*Betaproteobacteria*	*Ramlibacter ginsenosidimutans* strain BXN5-27	EU423304	96
IAD-9	*Actinobacteria*	*Nocardioides halotolerans* strain MSL-23	EF466122	98
IAD-10	*Bacteroidetes*	*Spirosoma fluminis* strain 15J17	LC148305	91
IAD-11	*Alphaproteobacteria*	*Sphingopyxis wooponensis* strain 03SU3-P	HQ436493	94
IAD-12	*Actinobacteria*	*Microbacterium saccharophilum* strain K-1	AB736273	97
IAD-13	*Actinobacteria*	*Nocardioides halotolerans* strain MSL-23	EF466122	98
IAD-14	*Alphaproteobacteria*	*Methylobacterium dankookense* strain SW08-7	FJ155589	97
IAD-15	*Actinobacteria*	*Nocardioides soli* strain mbc-2	JF937914	93
IAD-19	*Alphaproteobacteria*	*Sphingopyxis wooponensis* strain 03SU3-P	HQ436493	95
IAD-21	Candidate division FBP	*Oscillibacter valericigenes* strain Sjm18-20 (650 bp)	AP012044	83
		*Egibacter rhizosphaerae* strain 80759 (1,472 bp)	KR605111	79
IAD-24	*Firmicutes*	*Staphylococcus hominis* subsp. *Novobiosepticus* strain GTC 1228	AB233326	99
IAD-28	*Bacteroidetes*	*Mucilaginibacter soli* strain R9-65	JF701183	96
IAD-29	*Alphaproteobacteria*	*Sphingomonas mucosissima* strain CP173-2	AM229669	99
IAD-30	*Actinobacteria*	*Microbacterium saccharophilum* strain K-1	AB736273	96
IAD-31	*Bacteroidetes*	*Fibrella aestuarina* strain BUZ 2	HE796683	86
IAD-32	*Alphaproteobacteria*	*Amaricoccus kaplicensis* strain Ben101	U88041	94
IAD-33	*Alphaproteobacteria*	*Sphingopyxis wooponensis* strain 03SU3-P	HQ436493	95
IAD-34	*Alphaproteobacteria*	*Sphingomonas asaccharolytica* strain Y-345	Y09639	98
IAD-37	*Alphaproteobacteria*	*Sphingomonas hankookensis* strain ODN7	FJ194436	99
IAD-41	*Actinobacteria*	*Cellulomonas pakistanensis* strain NCCP-11	AB618146	97
IAD-42	*Bacteroidetes*	*Spirosoma fluminis* strain 15J17	LC148305	91
IAD-43	*Actinobacteria*	*Cellulomonas pakistanensis* strain NCCP-11	AB618146	96
IAD-44	*Betaproteobacteria*	*Ramlibacter ginsenosidimutans* strain BXN5-27	EU423304	96
IAD-45	*Alphaproteobacteria*	*Methylobacterium brachythecii* strain 99b	AB703239	99
IAD-48	*Actinobacteria*	*Nocardioides halotolerans* strain MSL-23	EF466122	98

### Identification of isolates

The bacterial cells suspended in TE buffer for DNA extraction were thawed, added to 20 μl of phenol:chloroform:isoamyl alcohol (25:24:1; Wako, Monza, Lombardy), and mixed by vortexing for 30 s to lyse the bacterial cells. The lysed cells were clarified by centrifugation at 15,000 rpm for 5 min, and one μl of supernatant was used as template for PCR. Template DNA was amplified with an iCycler (Bio-Rad Laboratories, Inc., Hercules, CA, USA) using 0.25 μl of TaKaRa Ex Taq (five U/μl), five μl of 10× Ex Taq Buffer, four μl of dNTP mix (Takara, Kusatsu, Japan), and 50 pmol of primers 8F (Weisburg et al., 1991; 5′-AGA GTT TGA TCM TGG CTC AG-3′) and 1492R (Lane, 1991; 5′-TAC GGY TAC CTT GTT ACG ACT T-3′) in a 50-μl reaction. The thermal cycling profile was one cycle at 94 °C for 20 s, 30 sequential cycles of 94 °C for 20 s, 55 °C for 30 s and 72 °C for 1 min, followed by a final extension at 72 °C for 7 min and a hold at 4 °C. The PCR products were purified using a QIAquick PCR Purification Kit (QIAGEN, Hilden, Germany). The concentration of the purified DNA was determined using a V-730BIO Spectrophotometer (JASCO Corporation, Tokyo, Japan). A 100-ng aliquot of PCR-amplified DNA and 7.5 pmol of 8F primer were mixed in a total volume of 15 μl and analyzed by Sanger sequencing by Takara Bio Inc. (Kusatsu, Japan). In general, the sequencing was performed with a BigDye Terminator v3.1 Cycle Sequencing Kit (Takara, Kusatsu, Japan) and an Applied Biosystems 3730xl DNA Analyzer (Thermo Fisher Scientific Inc., Waltham, MA, USA). The sequence reads obtained were compared with those in the NCBI database of rRNA type strains/prokaryotic 16S ribosomal RNA (database of bacterial and archaeal type strains, except environmental clones, hereafter referred to as type strains) using the BLAST program. Taxonomic classification at the genus level was performed using RDP Classifier as previously described (Wang et al., 2007).

### Phylogenetic analysis of strain IAD-21

BLAST searches of partial 16S rDNA sequences indicated that the sequence similarity of the strain IAD-21, isolated from DR2A medium, with type strains in the database was extremely low at 83%. The molecular phylogenetic analysis based on 16S rDNA sequence for strain IAD-21 was performed by TechnoSuruga Laboratory Co., Ltd. (Shizuoka, Japan). The DNA was extracted from the IAD-21 bacterial cells using a crude preparation of the lytic enzyme Achromopeptidase^®^ (Wako, Monza, Lombardy) and PCR amplified using PrimeSTAR HS DNA Polymerase (Takara, Kusatsu, Japan) with primers 9F (5′-GAG TTT GAT CCT GGC TCA G-3′) and 1541R (5′-AAG GAG GTG ATC CAG CC-3′) (Nakagawa & Kawasaki, 2001). Sequencing was performed using a BigDye Terminator v3.1 Cycle Sequencing Kit (Applied Biosystems, Waltham, MA, USA) and an ABI PRISM 3130 xl Genetic Analyzer System (Applied Biosystems, Waltham, MA, USA) with primers 9F, 785F (5′-GGA TTA GAT ACC CTG GTA GTC-3′), 802R (5′-TAC CAG GGT ATC TAA TCC-3′), and 1541R. The precise nucleotide sequence was determined with ChromasPro 1.7 (Technelysium, South Brisbane, QLD, Australia). The full-length reads of 16S rDNA sequence obtained (about 1,500 bp) were compared with sequences in DB-BA 12.0 (TechnoSuruga Laboratory Co., Ltd., Shizuoka, Japan) and international nucleotide sequence databases, including the DNA Data Bank of Japan, the European Nucleotide Archive, and GenBank (DDBJ/ENA(EMBL)/GenBank) using the TechnoSuruga Lab Microbial identification system (TechnoSuruga Laboratory Co., Ltd., Shizuoka, Japan). Since the full length of the 16S rDNA sequence of strain IAD-21 showed high similarity with clones derived from candidate division FBP (Lee et al., 2013), some 16S rDNA sequences of candidate division FBP and some bacterial phyla (*Actinobacteria*, *Armatimonadetes*, *Chloroflexi*, and candidate division WS1) were obtained from the database and subjected to molecular phylogenetic analysis (Table S2). Following multiple-sequence alignment by CLUSTAL W (Thompson, Higgins & Gibson, 1994), the alignment was edited with BioEdit, version 7.2.5 (Hall, 1999). A phylogenetic tree was constructed using the neighbor-joining (Saitou & Nei, 1987) method. The Kimura 2-parameter model for estimating nucleotide substitutions (Kimura, 1980) was employed using the molecular evolutionary genetics analysis (MEGA) software, version 6.0 (Tamura et al., 2013). The maximum likelihood (Felsenstein, 1981) method was employed using the Tamura–Nei model (Tamura & Nei, 1993) and MEGA ver 7.0 (Kumar, Stecher & Tamura, 2016). Bootstrap values (Felsenstein, 1985) were determined from 1,000 re-samplings.

### Nucleotide accession number

Culture-independent MiSeq sequence reads of the 16S rDNA have been deposited in the DDBJ sequence read archive (DRA) under accession numbers DRA006430 and DRA008228. Sequence reads of 16S rDNA from the bacterial isolates have been deposited in the DDBJ nucleotide sequences databank under accession numbers LC361357–LC361426.

## Results

**Figure 1:**
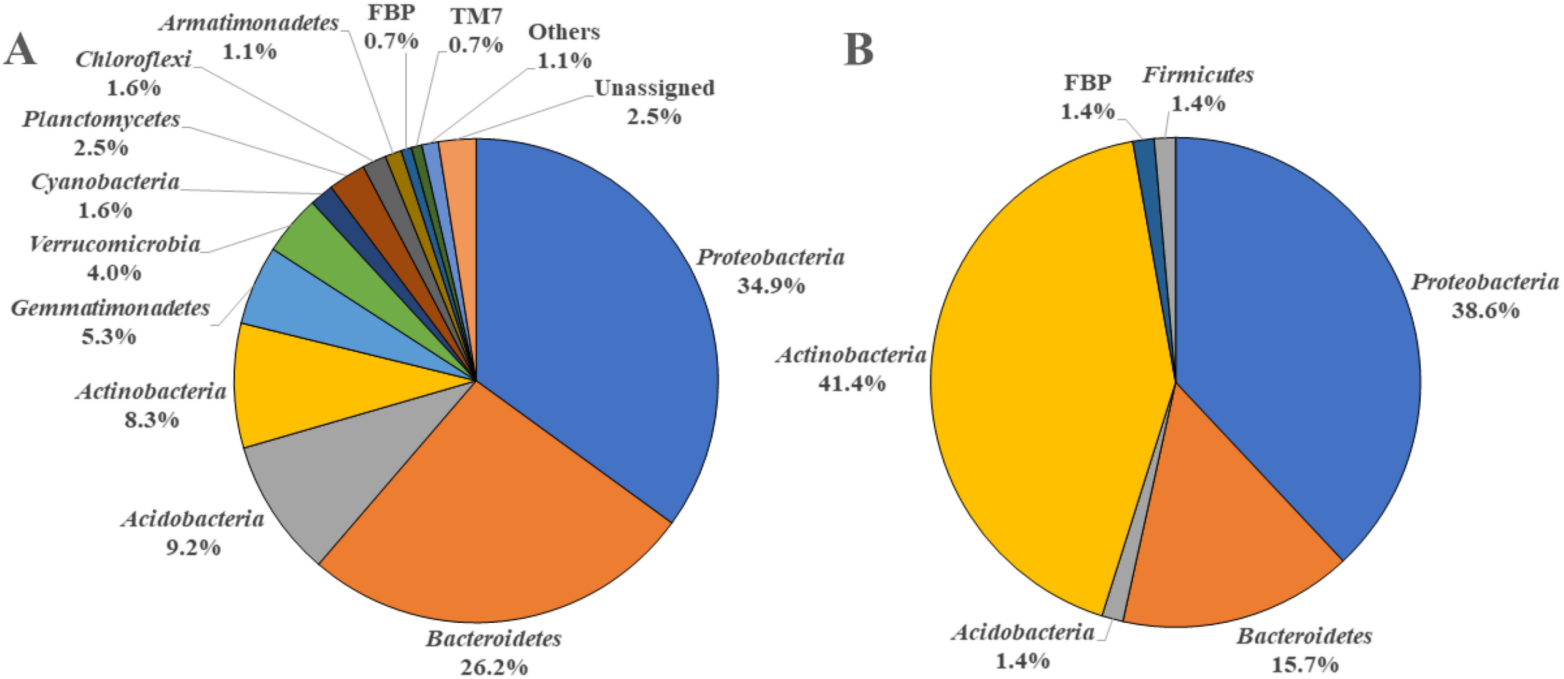
Bacterial phyla detected from *A. palmatum* bark. Relative abundances of bacterial phyla detected from *Acer palmatum* bark. (A) Results from the analysis of 16S rDNA sequences detected by culture-independent evaluation of bark using next generation sequencing with a MiSeq system. (B) Isolates obtained by culture-dependent analysis using PE03 and DR2A agar media, and sequenced by Sanger method followed by sequence alignment and characterization analyses.

**Figure 2:**
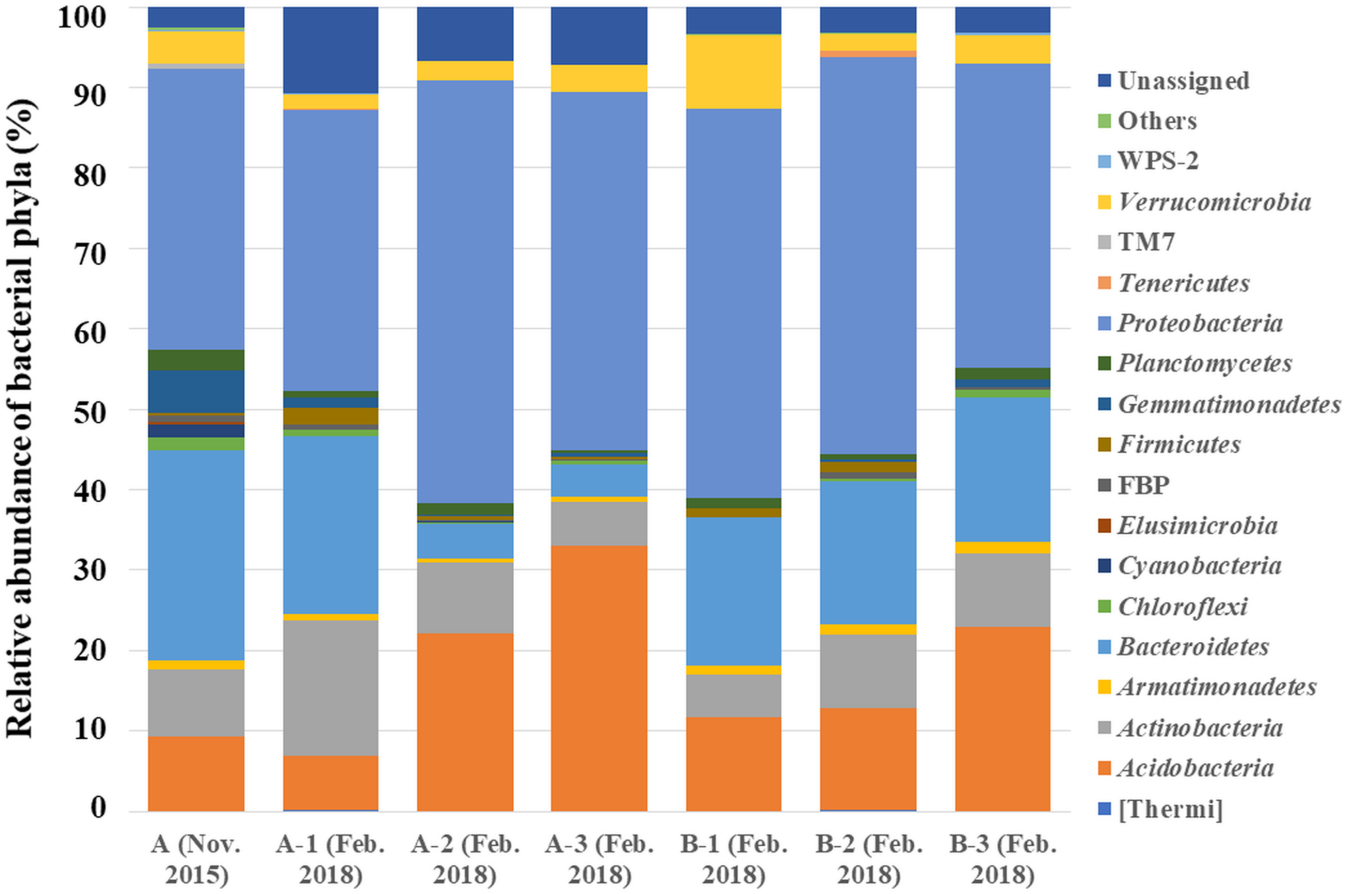
Bacterial phyla detected from seven samples collected from two *A. palmatum* trees. Relative abundances of bacterial phyla detected from seven samples collected from two *A. palmatum* trees. Results from the analysis of 16S rDNA sequences detected by culture-independent evaluation of bark using next generation sequencing with a MiSeq system. A and B refer to the tree number, and 1–3 refer to biological replicates within a single tree. Sample A (Nov. 2015) is identical to that in Fig. 1A.

By analyzing the microbial community structure of *Acer palmatum* bark using MiSeq, a total of 97,288 reads were detected. In total, 4,560 OTUs were defined with 97% sequence similarity. The phylogenetic distribution of the defined OTUs at the phylum level is shown in Fig. 1A. Sequence reads belonging to 27 bacterial phyla were detected from the *Acer palmatum* bark. *Proteobacteria*, at 34.9%, was the most abundant bacterial phylum, followed by 26.2% for *Bacteroidetes*, and 9.2% for *Acidobacteria*. In addition, *Gemmatimonadetes* (5.3%), *Verrucomicrobia* (4.0%), and *Armatimonadetes* (1.1%) were also detected, of which many bacteria were uncultured. Candidate division FBP (0.7%) was also detected at a relatively low abundance. In order to confirm whether the above phyla could be universally detected from *Acer palmatum* bark, three samples were collected from two individual trees (one of them being the previously analyzed tree), and re-analysis of the 16S amplicon sequencing was performed (Fig. 2; Table S3). A total of 51,109–77,943 reads were detected, and 673–1,794 OTUs were defined with 97% sequence similarity. These phyla (*Acidobacteria*: 6.7–33.1%, *Gemmatimonadetes*: 0.1–1.3%, *Verrucomicrobia*: 1.9–9.1%, *Armatimonadetes*: 0.4–1.4%) and candidate division FBP (0.04–0.7%), although varying in abundance, were also detected in the re-analysis (Fig. 2). Consistently, *Proteobacteria* was the most abundant phylum in all samples, although the ranks in the lower abundances were quite variable. Thus, both candidate divisions and rarely cultivated groups were found in the *Acer palmatum* bark.

The top 30 genera, found in the *Acer palmatum* bark based on MiSeq analysis, are shown in Table S3. *Sphingomonas*, *Actinomycetospora*, unidentified genus in *Chitinophagaceae*, unidentified genus in *Sphingomonadaceae*, unidentified genus in *Methylocystaceae*, and unidentified genus in *Acetobacteraceae* were commonly detected in the top 30 genera in all seven samples. It was suggested that these genera universally inhabit *Acer palmatum* bark. Furthermore, some genera belonging to phyla *Acidobacteria*, *Verrucomicrobia*, *Gemmatimonadetes*, and *Armatimonadetes* were among the top 30 at the genus-level in terms of relative abundance. These results revealed that many uncultured bacteria inhabited the *Acer palmatum* bark.

To determine whether these microorganisms could be cultured and isolated, we incubated *Acer palmatum* bark for 2 weeks in DR2A or PE03 agar medium. A large number of colonies were obtained on both media. Among the 96 isolated strains (48 strains isolated from each medium), there were nine strains from the PE03 medium and 17 strains from the DR2A medium that could not be sub-cultured. The remaining 39 strains from the PE03 medium and 31 strains from the DR2A medium could be sub-cultured and were subjected to sequence analysis. The phylogenetic distribution of the isolated strains at the phylum level is shown in Fig. 1B. Of the isolated strains, *Actinobacteria* was the most frequent with 29 strains (41.4%), followed by *Proteobacteria* with 27 strains (38.6%), and *Bacteroidetes* with 11 strains (15.7%). Compared with the results of MiSeq analysis, the results from the isolation analysis differed in the relative abundance at the phylum level. Compared with the top 30 genera detected by MiSeq analysis from the seven samples (Table S3), the genera that could be cultured and isolated had three genera in common (*Sphingomonas*, an unidentified genus in *Sphingomonadaceae*, and *Actinomycetospora*). Genus-level relative abundance was low for most of the cultured and isolated genera detected in the bark. For example, the relative abundances of *Novosphingobium* (0–0.03%) and *Nocardioides* (0.001–0.21%) in the *Acer palmatum* bark were very low. The results of culture-independent analysis do not always accurately reflect the actual microbial community structure in the bark due to variation among taxa in DNA extraction efficiency, 16S copy number variation and bias of universal primers. However, this suggested that whether microorganisms in the bark could be cultured or not was not predictable based on the relative abundance in the bark.

The classification at the genus level of all isolated strains obtained on the two types of medium is shown in Table 3. *Sphingomonas* was the most frequently isolated genus in this study with nine strains, followed by *Microbacterium* with five strains, *Spirosoma* with four strains, an unclassified genus in *Cellulomonadaceae* with four strains, and an unclassified genus in *Nocardioidaceae* with four strains. In addition, a bacterial strain belonging to *Acidobacteria*, which is difficult to culture and has very few isolated strains (Eichorst, Kuske & Schmidt, 2011; Navarrete et al., 2013; Tanaka et al., 2017), was isolated on PE03 medium, and based on classifier and BLAST analysis was presumed to belong to the genus *Terriglobus*.

**Table 3:** Taxonomic classification of isolates obtained by culture-dependent analysis on the basis of classifier program.

Phylum	Class	Order	Family	Genus	Number of isolates	
					PE03	DR2A
*Proteobacteria*	*Alphaproteobacteria*	*Caulobacterales*	*Caulobacteraceae*	Unclassified	2	
		*Rhizobiales*	*Bradyrhizobiaceae*	Unclassified	2	
			*Methylobacteriaceae*	*Methylobacterium*		2
			Unclassified	Unclassified	1	
		*Rhodobacterales*	*Rhodobacteraceae*	Unclassified		1
		*Sphingomonadales*	*Sphingomonadaceae*	*Novosphingobium*	2	
				*Sphingomonas*	6	3
				Unclassified		3
	*Betaproteobacteria*	*Burkholderiales*	*Comamonadaceae*	*Ramlibacter*		1
				*Variovorax*	2	
				Unclassified		1
	*Gammaproteobacteria*	*Rhodospirillales*	Unclassified *Rhodospirillales*	*Enhydrobacter*	1	
*Bacteroidetes*	*Cytophagia*	*Cytophagales*	*Cytophagaceae*	*Fibrella*		1
				*Spirosoma*	1	3
				Unclassified		1
			*Hymenobacteraceae*	*Hymenobacter*	1	
	*Flavobacteriia*	*Flavobacteriales*	*Flavobacteriaceae*	*Flavobacterium*	1	
	*Sphingobacteriia*	*Sphingobacteriales*	*Sphingobacteriaceae*	*Mucilaginibacter*	1	2
*Acidobacteria*	*Acidobacteriia*	*Acidobacteriales*	*Acidobacteriaceae*	*Terriglobus*	1	
*Actinobacteria*	*Actinobacteria*	*Actinomycetales*	Unclassified	Unclassified	2	
		*Corynebacteriales*	*Mycobacteriaceae*	*Mycobacterium*	1	
		*Micrococcales*	*Cellulomonadaceae*	Unclassified	2	2
			*Microbacteriaceae*	*Amnibacterium*	3	
				*Lysinimonas*	1	
				*Microbacterium*	3	2
				Unclassified	2	1
		*Propionibacteriales*	*Nocardioidaceae*	*Nocardioides*		1
				Unclassified		4
			*Propionibacteriaceae*	*Microlunatus*	1	
		*Pseudonocardiales*	*Pseudonocardiaceae*	*Actinomycetospora*	2	1
				*Pseudonocardia*	1	
FBP	Unclassified	Unclassified	Unclassified	Unclassified		1
*Firmicutes*	*Bacilli*	*Bacillales*	*Staphylococcaceae*	*Staphylococcus*		1
Total isolate number					39	31

**Figure 3:**
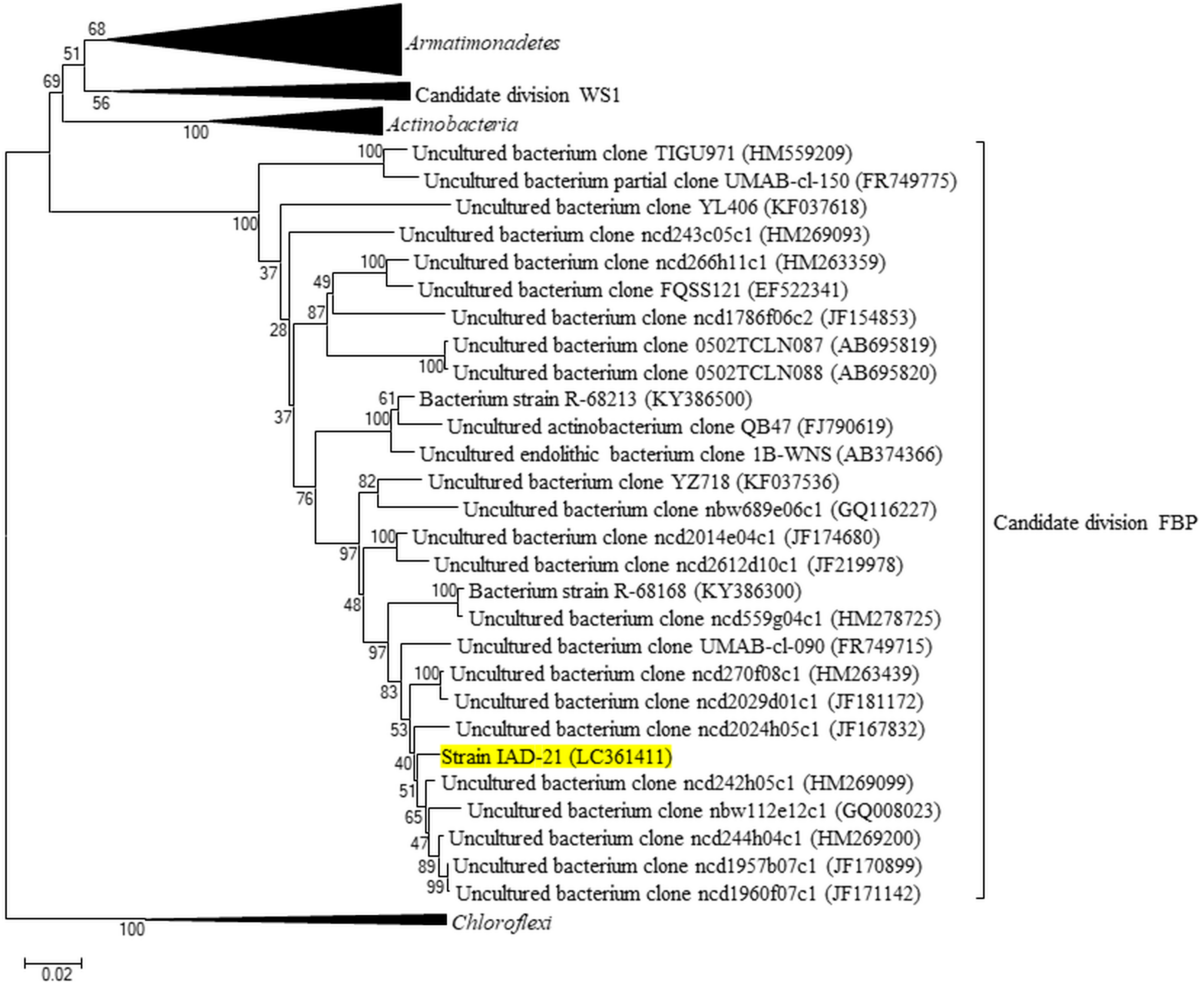
Phylogenetic tree of strain IAD-21. Phylogenetic tree of strain IAD-21 and related sequences of candidate division FBP based on 16S rDNA. In part, the full-length reads of 16S rDNA sequences were compared to sequences in international nucleotide sequence databases including the DNA Data Bank of Japan, the European Nucleotide Archive, and GenBank (DDBJ/ENA/GenBank). The phylogenetic tree was constructed using the neighbor-joining method and the Kimura 2-parameter model for estimating nucleotide substitutions. Bootstrap values were determined from 1,000 re-samplings. The newly identified and unique strain IAD-21 is located within the candidate division FBP cluster. The scale is given below the phylogenetic tree.

According to Tamaki et al. (2009), the isolates were phylogenetically divided into two groups on the basis of their partial 16S rDNA sequence similarities to the reference sequences in the public databases: (i) ≤97% similarity to type strains: isolates with high phylogenetic novelty, and (ii) >97% similarity: isolates with low phylogenetic novelty. These criteria were used as objective indicators of the phylogenetic novelty of isolates, although they do not necessarily indicate taxonomic novelty at the genus or species level (Tamaki et al., 2009). The culture collection obtained from this study included 44 strains (62.8% of the total) that showed ≤97% 16S rDNA sequence similarity with type strains (Tables 1 and 2). In particular, a potentially novel microorganism, strain IAD-21, was isolated on DR2A medium (Table 2). The full-length 16S rDNA sequence for IAD-21 was determined, and its sequence similarity with type strains was confirmed. Strain IAD-21 showed the highest similarity of 79% with *Egibacter rhizosphaerae* strain 80759 (Accession number KR605111) (Table 2). We conducted a BLAST search including environmental clones and found high sequence similarity with clones belonging to candidate division FBP, including clone UMAB-cl-090 obtained from the Antarctic soil (sequence similarity 95.2%; accession number FR749715), clone ncd242h05c1 obtained from human volar forearm skin (sequence similarity 97.2%; accession number HM269099), and clone ncd1960f07c1 obtained from human antecubital fossa skin (sequence similarity 96.9%; accession number JF171142) (Lee et al., 2013). Since the full length of the 16S rDNA sequence from strain IAD-21 showed the highest sequence similarity with a bacterial strain belonging to *Actinobacteria* (*E. rhizosphaerae* strain 80759), we obtained 16S rDNA sequences from *Actinobacteria*, and from *Armatimonadetes* and *Chloroflexi*, which are considered to be phylogenetically close to *Actinobacteria*. Sequences of the top 100 hits from the BLAST search of strain IAD-21, and sequences used for generating the phylogenetic tree of candidate division FBP and WS1 from Lee et al. (2013), were subjected to molecular phylogenetic analysis (Table S2). As a result, the cluster containing strain IAD-21 was determined to be phylogenetically separate from the known bacterial taxa, and it was suggested that strain IAD-21 belonged to the same cluster as sequences from the candidate division FBP (Fig. 3).

## Discussion

Although many studies are being carried out on the microbial community structure associated with the tree phyllosphere or rhizosphere, there are few reports that focus on the microbial community structure existing in the bark. In this study, we performed culture-independent analysis using MiSeq and isolation experiments to investigate the microbial community structure existing in *Acer palmatum* bark.

As a result of cultivation and isolation experiments, *Microbacterium*, an unclassified genus in *Cellulomonadaceae*, an unclassified genus in *Microbacteriaceae*, an unclassified genus in *Nocardioidaceae* (all belonging to *Actinobacteria*), and *Sphingomonas* were frequently isolated in this study. Microorganisms from numerous closely related genera have also been detected in culture-dependent and -independent analyses of other barks and branches including elm, poplar, grapevine, *Acer negundo*, *Acer pseudoplatanus*, and *G. biloba* (Mocali et al., 2003; Ulrich, Ulrich & Ewald, 2007; Martins et al., 2013; Shen & Fulthorpe, 2015; Leff et al., 2015; Aschenbrenner et al., 2017), and are considered to be the natural inhabitants of bark. To the best of our knowledge, there are no prior reports of *Spirosoma* being cultured and isolated from bark. Since the chemical constituents of the bark differ depending on the tree species (Feng et al., 2013), it is considered that the bark of different tree species would have different microbial communities. In the future, more detailed analysis of microbial community structure in the bark will be required with respect to changes in the community depending on the tree species and localization of microorganisms in bark organs, using both culture-dependent and -independent analyses. Through the current culture-dependent analysis, a wide range of microbial species was identified.

By culture-independent analysis of *Acer palmatum* bark, members of rarely cultivated phyla such as *Acidobacteria*, *Armatimonadetes*, *Verrucomicrobia*, and *Gemmatimonadetes* were detected. This is consistent with other culture-independent analyses of the microbial community structures of bark samples. For instance, *Acidobacteria* and *Verrucomicrobia* were detected in samples from *Acer pseudoplatanus* bark at frequencies of 10.7% and 4.0%, respectively (Aschenbrenner et al., 2017), and *Acidobacteria* and *Armatimonadetes* were detected from *G. biloba* bark samples at 13.1% and 1.0%, respectively (Leff et al., 2015). Since these exist universally in soil environments (Bergmann et al., 2011; DeBruyn et al., 2011; Lee, Dunfield & Stott, 2014; Kielak et al., 2016), it is believed that they are spread by means such as the wind or insects and colonize the bark. Although *Acidobacteria* are believed to be as environmentally widespread as *Proteobacteria* (Barns, Takala & Kuske, 1999), many of them are slow growing and oligotrophic bacteria that are largely comprised of uncultured taxa (Da Rocha, Van Overbeek & Van Elsas, 2009; Ward et al., 2009). Leff et al. (2015) suggested that old bark environments provide more suitable locales for stable inhabitation over long periods of time for slow-growing and oligotrophic bacteria such as *Acidobacteria* than do the leaf or branch environments. In addition, there is little disturbance from UV radiation or precipitation in the old bark environment. They cited this limited disturbance in the old bark as a factor for the richness of the microbial community in *G. biloba* bark and for the detection of rarely cultivated phyla. In a few reports from analyses of the microbial community structure of bark, it was stated that comparison of results from previous work was difficult due to the scarcity of available data (Leff et al., 2015). In reviewing the results from this study, we agree with this impression. While comparisons may be difficult, it is still possible to speculate. In addition, in the outer bark consisting of dead cells, light irradiation and symbiosis with lichens and cyanobacteria may occur, and in the inner bark consisting of living cells, flow of photosynthates may affect the symbiotic microbial community (Baldrian, 2017). It is possible that the long-term existence of these factors in a stable environment may promote the growth of a microorganism on the bark.

Martins et al. (2013) also reported the high diversity of acquired microorganisms in the bark as compared with that in organs such as leaves and fruits of grapevine. Recently in the bark tissue, microorganisms belonging to *Acidobacteria* (Yamada et al., 2014) and *Armatimonadetes* (Li, Kudo & Tonouchi, 2018) that are difficult-to-cultivate and slow-growing taxa were isolated. It can be inferred that the bark environment is a convenient residence for such bacterial taxa. Moreover, the fact that the bark harbors diverse microbial communities may have some meaning for trees. According to Khorsandy et al. (2016), the frequency of fungal endophytes in the bark of *Platanus orientalis* L. was significantly greater in older trees (60.04%) than in younger ones (39.96%). Existence of such fungal endophytes was positively correlated with the iron and potassium concentrations of the leaves, tree height, circumference, and improved visual appearance. These results suggested that fungal endophytes enhanced nutrient assimilation in trees, at least partly contributing to increased survival of the older trees (Khorsandy et al., 2016). Thus, there is no denying that old bark may benefit by harboring diverse microbial communities. However, since Khorsandy et al. (2016) reported fungal endophytes, while Leff et al. (2015) and Martins et al. (2013) referred to bacterial epiphytes, it is necessary to gain an understanding of the microbial community structure (both fungal and bacterial) of each organ of the bark area. It has been reported that members of *Acidobacteria* contribute to increases in biomass, rhizosphere morphology changes, production of indole-3-acetic acid, and iron absorption in *Arabidopsis thaliana* (Kielak, Cipriano & Kuramae, 2016). Further isolation and cultivation of microbes from phyla like *Acidobacteria*, which contain a considerable number of uncultured microbes, will lead to a better understanding of the tree-microbiota symbiotic system.

In this study, we successfully isolated a novel microorganism, strain IAD-21. Based on molecular phylogenetic analyses, it was suggested that IAD-21 belongs to candidate division FBP. In addition to bark, candidate division FBP has been detected by culture-independent analysis of Antarctic soil (Tytgat et al., 2016), with two strains belonging to this division isolated from Antarctic soil (Tahon & Willems, 2017). These strains were successfully isolated by mimicking the Antarctic environment, using a low-nutrient medium for phototrophic bacteria, and adjusting the photoperiod over 10 weeks. However, in the current study, strain IAD-21 was relatively easy to culture, as we succeeded in its isolation by simply using a general low-nutrient medium during a 2-week cultivation period. In addition, isolated strains from *Acer palmatum* bark were relatively novel, even if they belonged to taxa with high cultivation frequencies. The results of subsequent experiments exhibited the high phylogenetic novelty of isolates from *Acer palmatum* bark (Table S4). Furthermore, it is necessary to identify the reasons for obtaining high phylogenetic novelty of isolated strains and why strain IAD-21 could be cultivated with ease. One probable cause for the easy cultivation of strain IAD-21 could be its ability to grow in the relatively stable, less disturbed and unexplored environment of the bark. It is desirable that the culture efficiency be evaluated by the performance of comprehensive cultivation and isolation of microorganisms from the bark, and that the relationship between the poorly cultivated microorganisms and the tree bark be clarified.

## Conclusions

Based on our study, we propose that *Acer palmatum* bark might prove to be a promising source of novel microorganisms. Since the culture conditions used in this study were relatively non-exceptional and only involved using low-nutrient media, it is possible that additional microorganisms could be acquired utilizing special culture methods and conditions (e.g., modification of the culture substrate, gelling agents, and medium composition). Analysis of the microbial community structure of various tree species and the isolation of uncultured microorganisms may lead to a more comprehensive understanding of the yet uncharacterized tree-microbiota symbiotic system. Microorganisms from bark samples may also be important from an academic point of view to understand microbial ecology, and further research is expected to clarify the unknown sectors of the microbial phylogenetic tree.

## Supplemental Information

10.7717/peerj.7876/supp-1 Supplemental Information 1 Analysis workflow and QIIME parameter settings used in this study. Analysis workflow and QIIME parameter settings used in this study. QIIME version 1.9.0 (http://qiime.org/1.9.0/) was used in this study.These commands (from gunzip to filter_fasta.py) were used for each MiSeq run file.

10.7717/peerj.7876/supp-2 Supplemental Information 2 Sequences used for phylogenetic analysis.

10.7717/peerj.7876/supp-3 Supplemental Information 3 Percentage of top 30 genera detected by culture-independent analysis from *Acer palmatum* bark. Genus inferred to derive from chloroplast/mitochondria sequence and unassigned sequence was excluded from the list.

10.7717/peerj.7876/supp-4 Supplemental Information 4 Most similar sequences of isolated microbes from DR2A medium. Details of sampling procedure, isolation and identification of microbes are the same as the text except that the bark sample was collected in March 2019. The culture collection included 16/42 strains (38% of the total) that showed ≤97% 16S rDNA sequence similarity with valid species. Sequence reads of partial 16S rDNA from the bacterial isolates have been deposited in the DDBJ nucleotide sequences databank: LC490821–LC490862.

## Additional Information and Declarations

### DNA Deposition

The following information was supplied regarding the deposition of DNA sequences: Culture-independent MiSeq sequence reads of the 16S rDNA are available in the DDBJ sequence read archive (DRA): DRA006430 and DRA008228. Sequence reads of 16S rDNA from the bacterial isolates are available in the DDBJ nucleotide sequences databank: LC361357–LC361426 and LC490821–LC490862.

### Data Availability

The following information was supplied regarding data availability: The raw data are available as Supplemental Files.
