# Correction: Microbial community structure analysis in Acer palmatum bark and isolation of novel bacteria IAD-21 of the phylum *Abditibacteriota* (former candidate division FBP)

**DOI:** 10.7717/peerj.7876/correction-1

**Published:** 2020-04-04

**Authors:** Kazuki Kobayashi, Hideki Aoyagi

**Affiliations:** 1 Division of Life Sciences and Bioengineering, Graduate School of Life and Environmental Sciences, University of Tsukuba, Tsukuba, Ibaraki, Japan; 2 Faculty of Life and Environmental Sciences, University of Tsukuba, Tsukuba, Ibaraki, Japan

Corrigendum:

After publication, a reader notified the authors of certain issues regarding the bacteria taxonomy and the topology of the phylogenetic tree. Accordingly, the following changes have been made:

Candidate division FBP has been now named as *Abditibacteriota* (Tahon et al. 2018). *Abditibacterium utsteinense* R-68213T is now officially described as a first-cultivated representative of *Abditibacteriota*. The taxonomic status “Candidate division FBP” has been replaced with *”Abditibacteriota”*, and strain R-68213T has been renamed to *Abditibacterium utsteinense* R-68213T (in the main article, Figures 1 - 3, Tables 2 and 3, Figure S1, Tables S2 and S3). The result of 16S rDNA similarity of strain IAD-21 has also been revised in the main article and in Table 2. 

Sequences belonging to *Actinobacteria* have been removed from the results of the phylogenetic tree because the authors report that the phylum is distant from the other groups. *Deinococcus-Thermus*, which is phylogenetically closer to *Abditibacteriota*, has been added as an outgroup (Table S2). Phylogenetic trees have been reconstructed by the authors using the Neighbor-joining method (the Kimura 2-parameter model) and the Maximum likelihood method (Tamura-Nei with G+I model) (Figure 3, Figure S1).

The use of shorter 16S rDNA (about ~600 bp) sequences for similarity search has been mentioned.

New references have been added, namely: Gu, Fu & Li (1995), Kumar et al. (2018) and Tahon et al. (2018).

Additionally, few references have been removed, namely: Kumar, Stecher & Tamura (2016) and Tamura et al. (2013).

