# Supplementary material for: Microbial community structure analysis in Acer palmatum bark and isolation of novel bacteria IAD-21 of the candidate division FBP
Source: PeerJ. 2019 Oct 29;7:e7876. doi: 10.7717/peerj.7876 (PMC6824334; doi:10.7717/peerj.7876)
Supplement: Supplemental Information 1 — Analysis workflow and QIIME parameter settings used in this study. QIIME version 1.9.0 (http://qiime.org/1.9.0/) was used in this study.These commands (from gunzip to filter_fasta.py) were used for each MiSeq run file. [file peerj-07-7876-s001.docx]

**Supplemental Table S1** Analysis workflow and QIIME parameter settings used in this study.

| Command | Options |
| --- | --- |
| gunzip | -c Sample_S##_L001_R#_001.fastq.gz > Sample.fastq |
| sed | -e “s/ #:N/_#_N /g” Sample .fastq > conv.fastq |
| fastx_barcode_splitter.pl | --bol --exact --bcfile [primer_f/r_list] --prefix  [split_prefix_f /r] |
| fastqExtracter.pl | [seqid_list_bothmatch_f/r] > [both_primer_match_fastq_f/r] |
| fastx_trimmer | -i [both_primer_match_fastq_f/r] -o [primer_trim_fastq_f/r]  -f 20 -l 250 |
| sickle | pe -f [primer_trim_fastq_f] -r [primer_trim_fastq_r]  -t sanger -o [quality_trim_fastq_f] -p [quality_trim_fastq_f]  -s [quality_trim_fastq_fr] -q 20 -l 130 |
| flash | [quality_trim_fastq_f] [quality_trim_fastq_r] -f 250 -r 230  -m 20 -o merged.fastq |
| fastq_to_fasta | -i [merged.fastq, reandlen=246-260] -o [converted_fasta] -n |
| usearch | -uchime_ref [converted_fasta] -db [GG13-8_97_otus.udb]  -strand plus -chimeras chimera.fasta |
| filter_fasta.py | -n -f chimera.fasta -s [chimera_ID_list] -o final.fasta |
| cat | *.fasta (all samples) > input.fas |
| pick_de_novo_otus.py | -i input.fas -o otus |
| biom | summarize-table -i otus/otu_table.biom -o otus/summary |
| summarize_taxa_through_plots.py | -i otus/otu_table.biom -o Taxa_summary -m map.txt -s  -c Description |
| biom | convert -i otus/otu_table.biom -o otu_table.txt  -table -type=”OTU table” -to-tsv -header-key taxonomy |

QIIME version 1.9.0 (http://qiime.org/1.9.0/) was used in this study.

These commands (from gunzip to filter_fasta.py) were used for each MiSeq run file.
