# Supplementary material for: Microbial community structure analysis in Acer palmatum bark and isolation of novel bacteria IAD-21 of the candidate division FBP
Source: PeerJ. 2019 Oct 29;7:e7876. doi: 10.7717/peerj.7876 (PMC6824334; doi:10.7717/peerj.7876)
Supplement: Supplemental Information 4 — Details of sampling procedure, isolation and identification of microbes are the same as the text except that the bark sample was collected in March 2019. The culture collection included 16/42 strains (38% of the total) that showed ≤97% 16S rDNA sequence similarity with valid species. Sequence reads of partial 16S rDNA from the bacterial isolates have been deposited in the DDBJ nucleotide sequences databank: LC490821–LC490862. [file peerj-07-7876-s004.docx]

**Supplemental Table S4** Most similar sequences of isolated microbes from DR2A medium.

Details of sampling procedure, isolation and identification of microbes were same as the text except that the bark sample was collected in March 2019. The culture collection included 16/42 strains (38% of the total) that showed ≤ 97% 16S rDNA sequence similarity with valid species. Sequence reads of partial 16S rDNA from the bacterial isolates have been deposited in the DDBJ nucleotide sequences databank under accession numbers LC490821–LC490862.

| Strain No. | Phylum | Most similar sequence | Accession No. | Similarity (%) |
| --- | --- | --- | --- | --- |
| IAD-49 | *Actinobacteria* | *Patulibacter brassicae* strain SD | KT581436 | 93% |
| IAD-50 | *Actinobacteria* | *Curtobacterium flaccumfaciens* strain BCCM/LMG 3645 | AJ312209 | 99% |
| IAD-51 | *Proteobacteria* | *Methylobacterium tardum* strain RB677 | AB252208 | 99% |
| IAD-52 | *Proteobacteria* | *Luteibacter yeojuensis* strain R2A16-10 | DQ181549 | 98% |
| IAD-53 | *Actinobacteria* | *Nocardioides exalbidus* strain RC825 | AB273624 | 98% |
| IAD-54 | *Actinobacteria* | *Nocardioides lianchengensis* strain D94-1 | HQ657322 | 97% |
| IAD-55 | *Proteobacteria* | *Luteibacter anthropi* strain CCUG 25036 | FM212561 | 98% |
| IAD-56 | *Actinobacteria* | *Curtobacterium oceanosedimentum* strain ATCC 31317 | GU269547 | 99% |
| IAD-57 | *Proteobacteria* | *Luteibacter anthropi* strain CCUG 25036 | FM212561 | 98% |
| IAD-58 | *Actinobacteria* | *Nocardioides halotolerans* DSM 19273 strain MSL-23 | EF466122 | 96% |
| IAD-59 | *Actinobacteria* | *Curtobacterium ammoniigenes* strain NBRC 101786 | AB266597 | 98% |
| IAD-60 | *Actinobacteria* | *Marmoricola aequoreus* strain SST-45 | AM295338 | 99% |
| IAD-61 | *Actinobacteria* | *Nocardioides szechwanensis* strain RHLT1-17 | JF750424 | 96% |
| IAD-62 | *Actinobacteria* | *Nocardioides exalbidus* strain RC825 | AB273624 | 98% |
| IAD-63 | *Proteobacteria* | *Caulobacter hibisci* strain THG-AG3.4 | KX263320.1 | 99% |
| IAD-64 | *Proteobacteria* | *Beijerinckia derxii* subsp. *venezuelae* strain DSM 2329 | AJ563934 | 95% |
| IAD-65 | *Actinobacteria* | *Frondihabitans sucicola* strain GRS42 | JX876867 | 97% |
| IAD-66 | *Actinobacteria* | *Mycobacterium intermedium* strain 1669/91 | X67847 | 96% |
| IAD-67 | *Proteobacteria* | *Methylobacterium phyllostachyos* strain BL47 | EU912444 | 98% |
| IAD-68 | *Proteobacteria* | *Sphingomonas alpina* strain S8-3 | GQ161989 | 99% |
| IAD-69 | *Proteobacteria* | *Methylovirgula ligni* strain BW863 | FM252034 | 97% |
| IAD-70 | *Proteobacteria* | *Methylobacterium trifolii* strain TA73 | FR847848 | 98% |
| IAD-71 | *Actinobacteria* | *Marmoricola aequoreus* strain SST-45 | AM295338 | 98% |
| IAD-72 | *Bacteroidetes* | *Chitinophaga sancti* strain NBRC 15057 | AB680762 | 98% |
| IAD-73 | *Bacteroidetes* | *Chitinophaga sancti* strain NBRC 15057 | AB680762 | 98% |
| IAD-74 | *Actinobacteria* | *Amnibacterium kyonggiense* strain KSL51201-037 | FJ527819 | 97% |
| IAD-75 | *Proteobacteria* | *Luteibacter anthropi* strain CCUG 25036 | FM212561 | 98% |
| IAD-76 | *Proteobacteria* | *Luteibacter anthropi* strain CCUG 25036 | FM212561 | 98% |
| IAD-77 | *Proteobacteria* | *Luteibacter anthropi* strain CCUG 25036 | FM212561 | 99% |
| IAD-78 | *Actinobacteria* | *Parviterribacter multiflagellatus* strain A22/0/F9_1 | KP981371 | 93% |
| IAD-79 | *Actinobacteria* | *Curtobacterium ammoniigenes* strain NBRC 101786 | AB266597 | 99% |
| IAD-80 | *Actinobacteria* | *Iamia majanohamensis* strain NBRC 102561 | AB360448 | 95% |
| IAD-81 | *Proteobacteria* | *Sphingomonas aerophila* strain 5413J-26 | KC735148 | 97% |
| IAD-82 | *Proteobacteria* | *Alsobacter metallidurans* strain SK200a-9 | AB231946 | 99% |
| IAD-83 | *Actinobacteria* | *Curtobacterium ammoniigenes* strain NBRC 101786 | AB266597 | 99% |
| IAD-84 | *Actinobacteria* | *Mycobacterium intermedium* strain 1669/91 | X67847 | 96% |
| IAD-85 | *Proteobacteria* | *Tardiphaga robiniae* strain R-45977 | FR753034 | 98% |
| IAD-86 | *Proteobacteria* | *Roseiarcus fermentans* strain Pf56 | KJ406703 | 96% |
| IAD-87 | *Actinobacteria* | *Parviterribacter multiflagellatus* strain A22/0/F9_1 | KP981371 | 94% |
| IAD-88 | *Actinobacteria* | *Iamia majanohamensis* strain NBRC 102561 | AB360448 | 95% |
| IAD-89 | *Actinobacteria* | *Marmoricola aequoreus* strain SST-45 | AM295338 | 99% |
| IAD-90 | *Proteobacteria* | *Mesorhizobium tamadayense* strain Ala-3 | AM491621 | 99% |
